# A possible new pathway in natural killer cell activation also reveals the difficulty in determining human NK cell function in cancer

**DOI:** 10.1186/s40425-018-0392-0

**Published:** 2018-08-07

**Authors:** Robert J. Canter, William J. Murphy

**Affiliations:** 1 Department of Surgery, Division of Surgical Oncology, University of California Davis Medical Center, Sacramento, CA 95817 USA; 2 Distinguished Professor of Dermatology and Internal Medicine, University of California Davis Medical Center, Sacramento, CA 95817 USA; 3 Departments of Dermatology and Internal Medicine UC Davis School of Medicine, University of California, Davis 2921 Stockton Blvd, Institute for Regenerative Cures, Room 1614, Sacramento, CA 95817 USA

**Keywords:** Natural killer cells, Platelet-derived growth factor, NKp44, TCGA, Immunotherapy

## Abstract

Immunotherapy is rapidly becoming the fourth arm of cancer treatment, and breakthrough successes have been observed in multiple malignancies. However, despite the potential for impressive anti-tumor effects, on average, only 25% of patients respond, and barriers clearly remain. Hence, uncovering innovative ways to apply immunotherapy and overcome immune resistance remains an unmet need in immuno-oncology. Natural killer (NK) cells are an attractive candidate for extending the promise of immunotherapy, although success to date has been largely limited to hematological cancers. An important study has identified novel ways in which NK cells sense and respond to tumors, and these findings may impact clinical translation of NK cells in cancer immunotherapy. Using the activating receptor NKp44, NK cells were shown to bind platelet-derived growth factor DD (PDGF-DD) which was secreted by tumors. Using transgenic mice, NKp44 binding of tumor-expressed PDGF-DD was able to limit tumor growth, and expression of natural cytotoxicity receptor-associated gene signatures (of which NKp44 is a member) was correlated to clinical outcomes. This study highlights the potential for effector-target interactions to impact immune homeostasis in previously unrecognized ways, while at the same time, underscoring the complexities inherent in pre-clinical/ translational experimental design which may confound clinical application of these interesting results.

## Main text

Immunologists tend to view immune cells principally in the context of immune-mediated signaling (i.e. cytokines or co-stimulatory/inhibitory ligands), and as such, we tend to overlook their general biology as cells residing in an organism responding to continuous cellular and molecular signals, particularly in non-immune tissues. Non-immune mediators such as growth factors, hormones, and adhesion molecules are all utilized by immune cells. Indeed, as immune cells can potentially reside anywhere, it makes sense that these non-immune pathways also play important, possibly critical, roles in immune function. Case in point, a recent study by Barrow et al. demonstrated that a platelet derived growth factor (PDGF) isoform is a putative ligand for the NK cell activating receptor, NKp44 or NCR2 [1]. NKp44 or NCR2 was initially characterized as an induced cell surface receptor on activated human NK cells (mice completely lack NKp44) which augmented cytolytic killing of tumor cells in vitro [2, 3]. As a member of the human natural cytotoxicity receptor (NCR) family, these different receptors (NKp44, NKp30, NKp46 among others) appear to transmit co-stimulatory signals to human NK cells and augment their function. It was previously reported that the ligand for NKp44 is a molecule called mixed lineage leukemia-5 (MLL5) which is a truncated protein observed on the surface of tumor cells [4] and later observed in HIV-infected T cells [5]. However, using a NKp44-GFP reporter system, Barrow et al. screened thousands of mouse and human proteins and demonstrated that PDGF-DD (a homodimer of activated PDGF which has A, B, C and D isoforms) can augment NK cell activation via NKp44 and induce production of cytokines such as interferon-gamma. Furthermore, supernatants of the activated NK cells inhibited tumor cell growth of a variety of human tumors in vitro [1]. Unfortunately, as mice have no homolog for NCR2 to ascertain in vivo function, generation of NCR transgenic mice to force the expression of human NKp44 on mouse NK cells was required to assess in vivo function. Subsequent challenge of these transgenic mice with a mouse melanoma line transduced with human PDGF-DD demonstrated that the NCR2-Tg mice could partially resist tumor challenge and these anti-tumor effects could be partially abrogated by blocking antibodies to NKp44. Finally, examining The Cancer Genome Atlas or TCGA, PDGF-D was observed in multiple human tumors and the presence of NCR2- activated gene expression “signatures” correlated with superior outcome in glioblastoma but not other cancers [1].

Several important conclusions can be drawn from this study. First PDGF-DD can stimulate activated NK cells by directly binding NKp44. Since NKp44 is only present on activated NK cells, it is important to recognize that clinical application using this approach will require prior NK activation. However, it is of interest that the authors focused on cytokine induction rather than CD107a detection as a readout of NKp44 triggering since previous publications have shown a dominant role of NKp44 in augmenting cytolytic capability [2], although this may be contingent on in vitro culture conditions and subsequent activation status of the NK cells. Similarly, despite using a high-throughput screen, the authors did not appear to demonstrate binding to the previously reported ligand for NKp44, MLL5 [4]. This may be due to variations in cell culture conditions, differential binding of NKp44 to cell surface molecules (such as MLL5) versus soluble proteins (such as PDGF-DD), or the use of reporter systems not contingent on additional protein interactions which can be a source of negative data when attempting to link to other studies. Given the strong biologic role of NK cells on viral resistance with profound effects on both viral and NK cell evolution (i.e. in response to cytomegalovirus) [6, 7], it seems logical that virally-infected cells also represent a source of possible targets for NK cell activating receptors and need to be assessed particularly in conjunction with other NK cell signaling molecules.

A critical issue in attempting to determine the possible in vivo relevance of the human NK cell effects observed in vitro are the striking species differences between mouse and human NK cells, which in the current study centers on the complete absence of NKp44 in mice. Tumor immunology necessitates in vivo validation and this presents a quandary given the numerous immunological differences between mouse and man [8]. For example, the ligands for another key NK activating receptor (NKG2D) differ in mice and humans. In humans, NKG2D binds the polymorphic MHC class I-like molecules MHC-I chain-related A (MICA) and MHC-I chain-related B (MICB), whereas in mice NKG2D binds to H-60 and Rae1β. Creating transgenic mice bearing the human molecules can provide a glimpse into putative in vivo roles (outside of xenogeneic modeling using human NK cells in immunodeficient mice). However, care must be undertaken in extrapolating the physiologic implications of these data as mouse NK cells have not evolved to utilize this protein or pathway. Another caveat concerns the transduction of mouse tumor lines with human proteins such as PDGF-DD. The lack of competing receptors or ligands may augment otherwise incidental effects (i.e. human NKp44 binding to human PDGF-DD), and one must always keep in mind that these are xenogeneic molecules expressed in an immunologically competent mouse. Finally, it is important to acknowledge that NCR2 expression in the TCGA database was not found to be significant with regard to glioblastoma outcome [1], in part due to lack of consistent NCR2 signal in the tumors assessed. Instead, genes associated with NKp44 activation (both cytokine and cell cycle gene clusters called “variates”) were indirectly found to correlate with greater survival in GBM. Although these genes are indeed associated with NKp44 activation, they are not “specific” for NK effector function, much less NKp44 triggering, as the majority of these cytokine and cell-cycle activation genes are also present on activated T cells. It would therefore be important to assess these derived signatures over a wide range of conditions before categorizing them as true “NKp44-specific” signatures. Thus, care must be taken before extrapolating the role of NK cells in general and NKp44 in particular with clinical outcome, particularly in the context of intricate, but potentially unrepresentative, animal models.

Yet despite these caveats, the study does open new avenues for further exploring the role of NK cells in cancer. It will be of particular interest to ascertain if the role of NK cells and/or NKp44 is heightened when oncolytic viruses are applied in GBM since NK cells can not only target the tumor but simultaneously limit viral efficacy. Further research is also needed to determine why the PDGF-DD isoform is specifically upregulated in some cancers and selected to be a target of NK cells. Finally, the study raises important questions on the increasing use of targeted therapies for possible unforeseen immune effects. PDGF inhibition has been targeted in cancer with some success [9, 10]. However, aside from the direct effects on the cancer, as the Barrow study highlights, this approach may unexpectedly result in inhibition of NK responses. The “net” effect is likely very dependent on the tumor type, the dependence on specific growth factor pathways, and whether NK cells play a role in resistance for specific tumors. Nonetheless, the study by Barrow et al. demonstrates that NK cells may have evolved to recognize heightened growth factor production as a possible “danger” signal. Whether this effect is strong enough to be applied in cancer therapy is unresolved, but given the complexities of NK cells and cancer biology, a careful assessment of the strengths and weaknesses of pre-clinical modeling is paramount when interpreting the possible clinical applications of innovative translational immuno-oncology studies.

## Abbreviations

GFP: Green fluorescent protein
HIV: Human immunodeficiency virus
MLL5: Mixed lineage leukemia 5
NCR: Natural cytotoxicity receptor
NK: Natural killer
PDGF: Platelet-derived growth factor
TCGA: The Cancer Genome Atlas
